# Evidence of practice gaps in emergency psychiatric care for borderline personality disorder: how can this be explained?

**DOI:** 10.1186/s12888-020-02892-7

**Published:** 2020-09-29

**Authors:** Cécile Cases, Stéphanie Lafont Rapnouil, Adeline Gallini, Christophe Arbus, Juliette Salles

**Affiliations:** 1 CHU Toulouse, Service de psychiatrie et psychologie, psychiatrie, F-31000 Toulouse, France; 2 CHU Toulouse, Service d’Epidémiologie, F-31000 Toulouse, France; 3 Inserm, UMR 1027, Epidémiologie et analyses en santé publique : risques, maladies chroniques et handicaps, F-31000 Toulouse, France; 4 Inserm Unité 1214 ToNIC, Toulouse NeuroImaging Center, Université Paul Sabatier, Toulouse, France; 5 Institut des Handicaps Neurologiques, Psychiatriques et Sensoriels- CHU de Toulouse, F-31000 Toulouse, France; 6 Inserm Unité 1043, Centre de Physiopathologie de Toulouse Purpan, Université Paul Sabatier, Toulouse, France; 7 Hôpital Purpan, Centre-hospitalo-universitaire de Toulouse, Nouveau bâtiment de Psychiatrie, 330, avenue de Grande-Bretagne, TSA 70034, 31059 Toulouse cedex 9, France

**Keywords:** Borderline personality disorder, Care recommendations, Emergency department

## Abstract

**Background:**

Recent research has highlighted that patients with borderline personality disorder (BPD) could experience symptomatic remissions. This led to the production of guidelines concerning the most appropriate care. In addition, as BPD patients frequently present at an emergency department (ED), specific recommendations concerning how they should be cared for there have also been developed. The recommendations include the referral of patients to inpatient, outpatient or specific crisis care. However, an issue that has not been addressed is the capacity of ED services to apply the care recommendations. The objective of our study, therefore, was to identify the factors limiting their use in the ED of Toulouse University Hospital.

**Methods:**

A panel of psychiatrists specializing in BPD care examined the medical files of 298 patients with a BPD diagnosis to determine which referrals were consistent or not, according to the care recommendations. A logistic regression was then performed to identify which sociodemographic, clinical, organizational or professional-training factors were associated with inconsistent referrals.

**Results:**

32% of patients experienced an inconsistent referral. Consultations performed during an on-call or day-off schedule were linked with inconsistent referrals, while an active follow-up was associated with the provision of consistent care.

**Conclusion:**

Changing how evaluations of BPD patients in the ED are organized during on-call and day-off schedules could improve the application of the care recommendations regarding the most appropriate referrals.

## Background

Borderline personality disorder (BPD) is a common psychiatric diagnosis, with a prevalence of 1 to 2% in the general population and 10 and 20% in the psychiatric out and inpatient populations, respectively [1, 2]. Historically, BPD was considered to be a chronic and debilitating condition that was less susceptible to improvement than other psychiatric disorders. This conception was, however, revised following longitudinal research over the last 4 years which demonstrated that the evolution of BPD is more complex. In particular, it was shown that symptomatic remissions, defined as the presence of less than two symptoms [3], are common in these patients. Indeed, remission was achieved in 25% of patients for 2 years [4]. Moreover, after 10 years of patient follow-ups, remissions of 2 months or more and 1 year were seen in 91 and 85% of patients, respectively [5]. These results highlighted that BPD symptoms can be improved, leading to the development of specific practice guidelines concerning how the condition should be treated, including a handbook produced by JG Gunderson - *Good Psychiatric Management for Borderline Personality Disorder* [6] - which is intended for use in clinical practice.

A stronghold for BPD care is the emergency department (ED). Indeed, individuals with the condition are an important cohort of its patients, with the Pascual et al. study [7] indicating that they account for 9% of total attendances. As a consequence, the ED is both a key link in the continuity of care for those with BPD and where crisis management can be performed [8]. The main objective of the care given to these patients in an ED is to alleviate suicidal and auto-aggressive behavior, although the means employed have to be adjusted appropriately because of possible iatrogenic effects [9, 10]. Indeed, it has been reported that inappropriate evaluations of the suicide risk, unjustified hospitalizations or the excessive use of medication could worsen the outcomes of these patients. Prolonged or repeat hospitalizations have notably been shown to both reinforce the ‘avoidance of real life’ attitudes of those with BPD and contribute to the non-regression of their symptoms. Moreover, having a BPD diagnosis comes with considerable stigma, with patients characterized as manipulative, attention-seeking and untreatable, leading to discriminatory practices in the delivery of healthcare services, including in the ED [11]. As a consequence, the NICE [12] and NHMRC [13] propose guidelines for BPD care. Moreover, Hong et al. proposed an approach for use by ED staff based on an adaptation of the *Good Psychiatric Management for Borderline Personality Disorder* handbook, making ED attendances more effective and less harmful for these patients [14, 15]. As we mentioned, recommendations for BPD management exist. However, we suggest that there are limitations that could prevent professionals from applying these recommendations correctly in clinical practice, especially regarding country culture, professional skills, and how care systems are organized [16]. It is critical to address these shortcomings, as resolving them would improve the implementation of the recommendations and, as a result, patient care. In our research, we aimed to identify the factors that may play a part in how the care recommendations for BPD treatment are used in the ED of Toulouse University Hospital (TUH). Therefore, we asked an expert panel to evaluate the care proposed for patients with BPD, using data collected about the characteristics of patients and clinicians, and about how relevant systems are organized.

## Methods

### Inclusion and exclusion parameters

We performed a retrospective study in the ED of TUH during the period between 5th January and 11th May, 2018. Data concerning patients over the age of 18 with a diagnosis coded as F 60.3 in the CIM-10 handbook [17] was extracted using the Urqual® software currently employed in the ED. We excluded patients with a diagnosis of intellectual disability (F70-F79), neurodevelopmental disorders (F80-F89), psychosis (F20-F29) or a neurodegenerative condition (G30-G32). This was done to standardize our analysis, and because these disorders, in our view, lead to specific behavioral problems that do not match the criteria for a diagnosis of BPD. Patients admitted in for justice requisition were also excluded from the analysis. The diagnosis was made by the resident psychiatrist or senior psychiatrist. The residents and the psychiatrists with less than 10 years of experience systematically benefited from a teaching of the Nice Guidelines [12] during their initial training. The psychiatrists with more than 10 year of experience were also trained for Nice Guidelines [12] but during continuous training. If a patient was admitted more than once in the six-month period, only the data relative to the first admission was analyzed.

### Primary outcome: consistency of the referrals, as determined by our expert panel

We had recourse to an expert panel of psychiatrists for our analysis of the data. This panel was composed of eight psychiatrists who are currently treating patients with BPD. Prior to the study, the panel also examined updates to the most recent research data relating to BPD patients in the ED, in particular, the NICE [12] and NHMRC [13] guidelines for BPD care. In addition, they also read the article from Hong et al. 2016. This article, being a perspective article based on an expert opinion, could not be considered as a guideline. Nevertheless, we considered it as valuable, since it brings pragmatic elements for psychiatrists working in ED and it is based on several valuable scientific publications about BPD care in ED. These data were sent to them in advance of the reading process of medical files. Then a meeting was programmed with the experts during which the guidelines and the reading process of the medical files was showcased in the presence of the research designers. However, after that, the experts did not benefit from any immediate reminder of those recommendations before their clinical evaluations. Each medical file was analyzed independently by two experts. In the case of disagreement between them, a third evaluation was conducted by a different member of the panel.

In order to ensure the consistency of the file read, the experts had to consider the following issues concerning each patient’s clinical circumstances: the care plan the socioenvironmental characteristics; and the reactivity of the structure to which the patient was referred. The experts also had to answer yes or no to the following question: “Was the proposed referral consistent?” This answer enabled us to categorize the patients into two groups: consistent and inconsistent referrals. The consistency was evaluated by the expert according to the recommendation that we previously mentioned as well as their clinical experience.

When working with the expert panel, we defined what could be considered as a consistent referral the following way:

- An inpatient referral is considered consistent for patients presenting true dangerousness (auto or hetero aggressive risk) with no possibility to organize an outpatient care by working with patients’ relatives. This consideration was elaborated with reference to the NICE guidelines [12].
- An outpatient referral to the usual care structure is considered consistent if the next appointments were already scheduled to occur within a short timeframe after the admission in ED. This consideration was elaborated with reference to the NICE guidelines [12] that mentioned to “offer a follow-up appointment at an agreed time”. Moreover, we classified such referral as consistent if a transmission was made between ED and the usual care structure to inform of the admission in ED.
- An outpatient referral to a crisis structure is considered consistent if the patient did not already benefit from active psychiatric follow up. Indeed, the NICE guidelines [12] mentioned that “transition from one service to another, may evoke strong emotions and reactions in people with borderline personality disorder” and that “changes are discussed carefully beforehand with the person (and their family or careers if appropriate) and are structured and phased”. For this reason, we considered that is was not consistent to refer the patient to a new ward if the patient already benefits from a psychiatric follow-up. Considering crisis structures, they are indeed strongly connected to the ED and propose an intensive care in order to foster the therapeutic alliance for patients initiating psychiatric care. Moreover, the transmission of information between ED and crisis structures is systematic and these structures propose psychiatric appointments within 2 days following the ED admission. Indeed, the NHMRC guidelines [13] mentioned that “Health professionals within each type of service should set up links with other services to facilitate referral and collaboration.”

Conversely, we considered as an inconsistent referral a hospitalization that was decided without first exploring alternative solutions involving the social support of the patient. Indeed, the NICE guidelines [12] mentioned to “explore other options before considering admission to a crisis unit or inpatient admission”. For outpatient care, inconsistent referrals can be either a future appointment that has not been clearly organized (e.g. only a phone number given to the patient), or that has been organized with a long waiting time.

Finally, the experts were asked: “Regarding the clinical element of the medical file, do you agree with the BPD diagnosis?” If both experts answered “no”, we excluded the patient from the analysis.

### Data

The data contained in the medical files consisted of:

- Medical characteristics, including: ongoing psychiatric follow-ups; a past history of outpatient and inpatient psychiatric care; current medication (mood stabilizers, anti-depressants, anti-psychotics, benzodiazepines); and psychiatric comorbidities (bipolar, depressive, anxiety, post-traumatic stress, eating and substance-use disorders).
- Sociodemographic characteristics, including: age; gender; family circumstances (single or not); whether there was a source of assistance; professional status (employed or not); and the receipt, or not, of social security benefits.
- ED admission characteristics, including: the reason for the admission. If this was attempted suicide, the method (hanging, self-poisoning, jumping from a window…) was specified, and a suicide intent score determined by the Pierce scale which is composed of three sub-scores was also collected, allowing us to collect data about objective circumstances, self-reports and the risk of a suicide attempt [18]. The time of the psychiatric assessment (night or day, defined by the on-call schedule); the day of the week (weekend or bank holiday versus a week day) was also collected. Indeed, in the psychiatric ED of the Toulouse University Hospital the consultations are delivered on days off, however the functioning of our unit is different on these days. During working days, five psychiatrists specialized in emergency psychiatry ensure the functioning of the ED, whereas during days off only one psychiatrist ensure it alone, without necessarily being accustomed to emergency psychiatry nor with adult psychiatry – it can also be a psychiatrist for child and adolescents. Finally, we also collected the medical experience of the professional who conducted the assessment (resident or senior).
- Referral: defined by the type referral proposed after the psychiatric evaluation. The TUH ED receives patients for psychiatric consultations and ensures that a psychiatric evaluation is performed after a suicide attempt. Patients can be discharged from the ED with a referral to various care services: 1) inpatient care, with the patient admitted to a psychiatric ward for a prolonged period of hospitalization; 2) outpatient care, which involves a psychiatric consultation with either a private psychiatrist or at a psychiatric center that employs professionals such as psychologists, nurses and social workers; and 3) a crisis structure, which also involves outpatient care, but with features like reactivity to appointment delays and short, intensive, and aggressive follow-ups. A crisis structure is a structure that ensures a quick appointment within 2 days following the admission in ED, and that ensures an intensive follow-up consisting of three appointments per week. In these structures the follow-up is short (6 weeks), based on brief therapy rationale in order to guarantee a turn over and thus the availability to welcome new patients. At the end of the follow-up the patient is referred to other care structures. This project is progressively built with the patient.

We determined the type of referral from the data and specified whether the patient had already been treated within this particular structure or not. We also established whether the crisis structure was accessible at the time of the attendance at the ED, as these wards can close to new admissions when the maximum number of patients they are able to treat is reached.

- Information on readmissions at 1 month was also collected, including the reasons for the readmission clustered by suicide attempt, suicide ideation, or other. In those data we examined if the patient was engaged in an active follow-up, defined as the fact that the patient attended her/his last appointment or if she/he did prevent the care team to program a future appointment if he could not attend it.

### Ethics

The use of the data was approved by the Comission Nationale de l’Information et des Libertés (CNIL), according to the French legislation MR-004.

### Statistical analysis

The patients’ baseline characteristics were determined. The continuous and categorical variables are described using the mean (+/− standard deviation) and/or median (+/− interquartile range), according to their distributions, and numbers and percentages, respectively. As there were more than two raters for some patients, we used the Fleiss kappa coefficient [19] to determine the inter-rater reliability of the evaluations. The prevalence of inconsistent referrals was then estimated and the 95% CIs calculated using the binomial exact method.

Associations between patients’ categorical characteristics and the appropriateness of the referrals were tested using the chi-squared or Fischer exact tests (when the expected values were less than five). A multivariable logistic regression was used to assess the link between the medical, sociodemographic and ED-admission characteristics and the consistency, or not, of the referral. The variables found to be significantly associated with the *p* < 0.2 bivariate analyses were included in the initial regression model, along with the potential confounding factors of age and suicide attempts. The model also included the following elements: age; gender; single or not; existence of an active medical or paramedical psychiatric follow-up; the use of seven psychotropic drug classes; psychiatric comorbidities; suicide attempt as the reason for the admission and the Pierce score; night or weekend admission; whether the crisis center was closed; and if the psychiatrist working in the ED was on call. We then performed a backwards step-by-step manual selection to produce our final model, controlling for confounding variables at each step. The statistical analyses were performed using the STATA® software, v14.2. Statistical significance was set at *p* < 0.05. We then tested the interactions between all the variables included in the final model.

## Results

### Population description

**Fig. 1:**
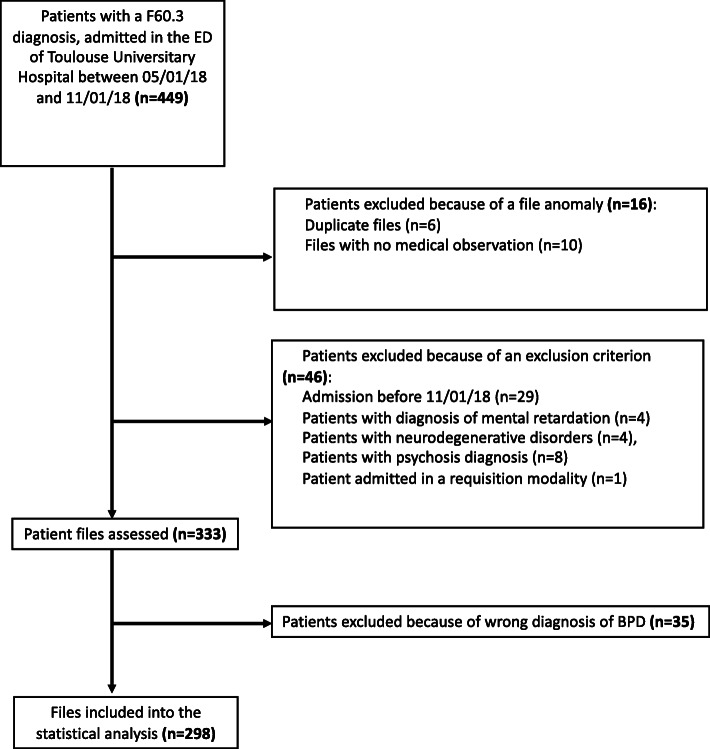
Flow chart

Of the 449 patients in our initial sample with a CIM-10 code of 60.3, 333 were evaluated by two experts on our panel, with both rejecting a BPD diagnosis for 35 of them; 298 patients were therefore ultimately included in the study. The flow chart is portrayed in detail in Fig. 1.

The mean age of the sample was 30.7 years (SD = 10.9); 74.2% were female, 65.1% were single, 48.7% were unemployed, 31.6% were in paid employment, 11.4% were students, and 19.1% received the social security allowance for adults with disabilities. Details of the sociodemographic characteristics are contained in Table 1.

**Table 1:** Socio-demographic characteristics

Sociodemographic characteristics	N	%
**Age**		
18–25	115	38.6
25–34	92	30.9
> 35	91	30.5
**Sex**		
Male	77	25.8
Female	221	74.2
**Family circumstances**		
Single	194	65.1
Couple	100	33.6
Unspecified	4	1.3
**Entourage**		
No source of support declared	32	10.7
Source of support declared	248	83.2
Unspecified	18	6.1
**Activity**		
No job	145	48.7
Employee	92	30.9
Self-employed	6	2.0
Student	34	11.4
Retired	2	0.7
Other	4	1.3
Unspecified	15	5.0
**Receipt of social assistance**		
None	118	39.6
Social-assistance pension	124	41.6
Unspecified	56	18.8

In terms of psychiatric comorbidities: 8.7% had a comorbidity for bipolar disorder; 14.4% for depressive episodes; 3.0% for anxiety disorders; 2.7% for post-traumatic stress disorder; and 9.7% for eating disorders. In terms of substance-use disorders, the numbers are as follows: 28.9% tobacco; 24.5% alcohol; 21.8% cannabis; 8.4% cocaine; and 2.0% opioids (2.0% of these patients were receiving maintenance treatment). In terms of treatment, 47.0% had not benefitted from active psychiatric follow-ups and 55.4% had already been hospitalized in a psychiatric ward. Concerning medication: 10.7% of patients were on mood stabilizers; 29.8% on antipsychotics (including first- and second-generation, and sedative antipsychotics such as cyamemazine); 41.3% on benzodiazepines; and 32.9% on antidepressants.

### Description of the ED admissions

The reasons for admission to the ED were: suicide ideation in 66.8% of cases; a suicide attempt in 40%; and non-suicidal self-injury, mostly cutting, in 8.7%. Some patients had several reasons for their admission, e.g., suicide ideation and a suicide attempt. The operative modes for the suicide attempts were: self-poisoning in 81.2% of cases; cutting in 9.3%; jumping from a window in 4.3%; and other in 5%. The mean Pierce score for the suicide-attempt population was 2.0 (SD = 4.0). Finally, 46.3% of patients were referred during a night- or on-call schedule, and 48.3% were evaluated by a senior psychiatrist.

### Referrals and their consistency

The proposed referrals were: outpatient care in 58.0% of cases (18.8% were referred for their first outpatient appointment and 39.2% to their usual outpatient care); crisis care in 12.4%; psychiatric hospitalization in 17.8%; and no specific referral in 10.1% of cases, as these patients chose to leave the ED without it.

In terms of the evaluations of the referrals’ consistency, a third expert was required for the cases of 65 patients (21.8%) and the inter-rater reliability was substantial (Fleiss kappa coefficient = 0.69 [19]; agreement = 85.5%).

**Fig. 2:**
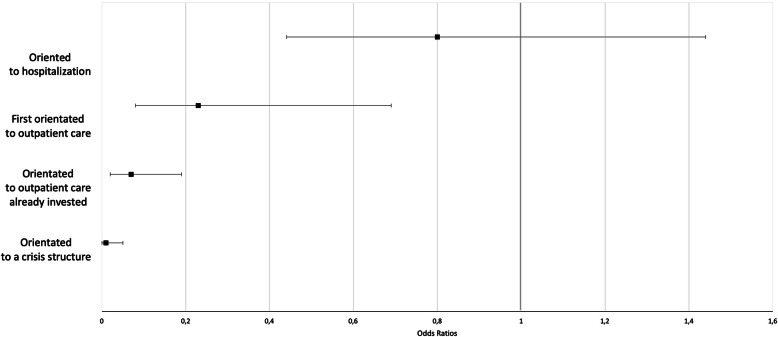
Forrest plot presenting the odds ratios for the inconsistency of the orientations. An OR under 1 indicates a reduction of the risk of inconsistency referral. Conversely, an OR over 1 indicates an increase of the risk for inconsistent referral

The experts determined that 36.2% (95% CI = [30.8–42.0]) of the referrals were inconsistent. The service to which patients were referred was associated with its consistency (*p* < 10^− 4^): referrals to a crisis center accounted for 19.0% of the consistent referrals and 0.9% of those that were deemed to be inconsistent. Referrals to other types of outpatient care was identified in 60.3% of the consistent referrals and 56.7% of those that were inconsistent. The numbers of consistent or inconsistent referrals to hospital care were similar: 18.0 and 18.3%, respectively. Finally, 24.0% of patients in the inconsistent-referral group were not referred to any specific care versus 2.6% in the consistent-referral group. Figure 2 summarizes the ORs relative to the type of referral.

### Factors associated with inconsistent referrals

In a multivariable analysis which controlled for age, gender, marital status and periods when the crisis centers were closed, we identified three factors independently associated with inconsistent referrals (Table 2): 1) no active psychiatric follow-up (*p* = 0.020); 2) no active follow-up by other professionals (nurse, psychologist) (*p* = 0.036); and 3) admissions at night or the weekend (OR = 1.95, 95% CI = [1.2–3.3]).

**Table 2:** Results for multivariate analysis. * Patients that benefit from a follow up by non-medical professional such as psychologist or nurses specialized in psychiatry

	Multivariate Analysis			
	adjusted OR	IC 95%		*p* value
Age				0.477
25–34	0.77	0.41	1.44	
35+	1.15	0.62	2.1	
Female	1.68	0.92	3.05	0.09
Not single	1.62	0.95	2.76	0.075
Medical follow-up	0.49	0.26	0.915	**0.02**
Paramedic follow-up*		0.53	0.95	**0.036**
Night or on-call admission	1.95	1.18	3.25	**0.01**
Crisis center closed	1.23	0.73	2.07	0.426

### Readmissions at one month

Among our population, 27.3% (95% CI = [22.3–32.7]) were readmitted to the ED at 1 month, 32.5% for suicide ideation, 33.8% for a suicide attempt and 33.7% for other reasons. The consistency of the referral was not significantly associated with readmission. Active follow-up was associated with a lower readmission risk; indeed, only 18.7% of the patients had benefitted from active follow-up in the readmitted group versus 81.3% in the other group (*p* = 0.008). The absence of a source of support was significantly associated with readmission: 75.7% of those who had such help were not readmitted to the ED at 1 month, versus 46.9% of those who did not (*p* = 0.001). An admission during an on-call or day-off schedule was also associated with more readmissions: 35.5% vs 20.3%, respectively (*p* = 0.003).

## Discussion

In our study, one third of the patients with a diagnosis of BPD who attended at TUH’s psychiatric ED experienced an inconsistent referral based on the current care recommendations.

Despite the retrospective data collection, the sociodemographic data of those in our sample was comparable to that obtained prospectively for BPD patients in the FRENCH CRISIS cohort. The majority of our patients were single women with a mean age of around 30 [20], while the main reasons for the attendances at TUH’s ED were mostly suicide attempts and suicide ideation, which is consistent with the relevant literature [21].

Our main finding was that the consistency of a referral was largely explained by the type of care structure to which the patients were oriented. Indeed, the referrals judged to be inconsistent mostly involved non-specific referrals. Conversely, referrals to high-level care, as provided by a crisis center for example, were systematically judged as consistent. These results support the fact that the definitive care for patient with BPD cannot be provided by the emergency department and that the care should be organized following a stepped care model wherein clinicians can provide acute services, followed by regular monitoring and follow-up, adapting different grades of intervention depending on treatment response. Indeed this model is based on the following beliefs: people should not have to wait for psychological service; different people require different levels of care; finding the right level of care often depends on monitoring outcomes; moving from lower to higher levels of care based on client outcomes often increases effectiveness and lowers costs overall [22]. In addition, this model has already been described and evaluated for patients with BPD and did show an improvement regarding suicide risk, symptom severity and quality of life as well as a reduction of the treatment cost [23–25].

The fact that the inconsistency of referral was mostly associated with nonspecific referral, including referrals with long waiting time for admission, and the fact that the lack of follow-up and support were major factor related to readmission, underline that the lack of availability of specific care structures is a major barrier to the implementation of the recommendations of care for BPD as well as the aforementioned stepped care model.

Other factors also had an impact. Evaluations performed during an on-call or day-off time period were associated with inconsistent referrals. We have not identified any earlier reports of such effects in the psychiatric-care literature, although their negative impact has been recorded in other medical fields (e.g., a higher mortality rate for patients with chronic obstructive pulmonary disease during weekend admissions [26]). In our study, it appeared that the higher number of inconsistent referrals in these circumstances could be explained by a lack of communication between the ED and downstream care structures. In addition, the fact that the patients without an active follow-up by such structures were mainly those who had experienced an inconsistent referral could support this hypothesis. This suggests that it is difficult for an ED to apply the BPD care recommendations in a population of patients who are not currently being supported in a care structure. Finally, we found that admissions during days off are associated with more inconsistent referrals. Indeed, during those days the medical team is reduced in number and thus less available to organize an outpatient care by working with patients’ relatives. Our study therefore showed that the lack of availability of human resources can significantly contribute to the lack of care recommendations implementation.

Interestingly, clinical features like comorbidities or the reasons for an admission did not have any impact on the use of the recommendations concerning appropriate referrals. Not have any impact on the use of the care recommendations concerning appropriate referrals. This was also true for a psychiatrist’s level of experience, suggesting that specific expertise is not required to apply the care recommendations in practice. Readmissions at 1 month were likewise not associated with the consistency of the referrals, but the lack of an active follow-up was. Patients evaluated during on-call or day-off time periods were also readmitted more at 1 month, which is relevant given that this group had experienced less consistent referrals. This raises an issue concerning patients who could be lost to care follow-ups, but investigating this was beyond the scope of this study.

Our research has some limitations, including: 1) its retrospective design and absence of new data collection; 2) the possible imprecision of the BPD diagnoses extracted from the CIM-10 codings, although the experts’ examinations of the medical files went some way towards improving this; and 3) its cross-sectional design, which only enabled us to identify associations and not test the explicative hypotheses 4) it was single site study.

Nevertheless, the study provides original data on the application of the BPD care recommendations in clinical practice in the ED. The barriers to their implementation can be put into three main categories: 1) the knowledge of the physicians (e.g., a lack of awareness and familiarity); 2) the attitudes of the physicians (e.g., a lack of agreement and/or motivation); and 3) external (e.g., patient-, guideline- and environment-related) [16, 27]. Environment-related factors were the main reasons why the recommendations for BPD care were not implemented in the ED in our study, with the most prominent being the care proposition; indeed, the type of care structure proposed was strongly associated with the consistency of the referral, although the fact that a downstream crisis center was closed was not significant.

The time of the ED attendances was important, with night or on-call admissions associated with less use of the care recommendations. This may be explained by changes to the organization of care in these periods; indeed, there were three times fewer psychiatrists working then than during the day. Moreover, at night and when on call, the ED psychiatrists had no opportunity to coordinate outpatient care. Given these findings, it is clear that care continuity must be improved, for example by asking patients to return to an ED or crisis center on a normal working day.

## Conclusion

Changing how evaluations of BPD patients in the ED are organized during on-call and day-off schedules could improve the application of the care recommendations regarding the most appropriate referrals.

## Data Availability

The datasets used during the current study are available from the corresponding author on reasonable request.

## Abbreviations

BPD: Borderline Personality Disorder
ED: Emergency Department
NICE: National Institute for Health and Care Excellence
NHMRC: National Health and Medical Research Council
TUH: Toulouse University Hospital
